# Supplementary material for: Polyketide synthase gene clusters in the ericoid mycorrhizal fungus Oidiodendron maius: genome-wide prediction, expression profiling, and functional characterization of knockout mutants
Source: Front Plant Sci. 2026 Sep 16;17:1924430. doi: 10.3389/fpls.2026.1924430 (PMC13623903; doi:10.3389/fpls.2026.1924430)
Supplement: Supplementary file 1 [file SupplementaryFile1.zip › Supplementary Materials/Supplementary Folder 1.zip!/Oidma1_AssemblyScaffolds_Repeatmasked/knownclusterblast/cluster34/jgi.p_Oidma1_204580_mibig_hits.html]

| MIBiG Protein | Description | MIBiG Cluster | MiBiG Product | % ID | % Coverage | BLAST Score | E-value |
| --- | --- | --- | --- | --- | --- | --- | --- |
| AGA37281.1 | methyltransferase | BGC0000819 | Paraherquamide\_biosynthetic\_gene\_cluster | 43 | 74.8031496063 | 249 | 6.9e-66 |
| AAC01738.1 | C-27\_O-methyltransferase | BGC0000136 | Rifamycin\_biosynthetic\_gene\_cluster | 30 | 54.3307086614 | 107 | 3.2e-23 |
| ABV97176.1 | Methyltransferase\_type\_11 | BGC0000137 | Rifamycin\_biosynthetic\_gene\_cluster | 31 | 55.6430446194 | 104 | 2.1e-22 |
| ACF35463.1 | MbcT | BGC0000090 | Macbecin\_biosynthetic\_gene\_cluster | 39 | 31.2335958005 | 104 | 3.6e-22 |
| ACF35464.1 | MbcU | BGC0000090 | Macbecin\_biosynthetic\_gene\_cluster | 41 | 30.7086614173 | 102 | 8e-22 |
| AAD28459.1 | MitM | BGC0000915 | Mitomycin\_biosynthetic\_gene\_cluster | 35 | 38.845144357 | 101 | 2.3e-21 |
| ACY01395.1 | O-methyl\_transferase | BGC0000177 | Dorrigocin\_/\_migrastatin\_biosynthetic\_gene\_cluster | 39 | 38.845144357 | 101 | 3e-21 |
| BAC55218.1 | methyltransferase | BGC0000825 | Staurosporine\_biosynthetic\_gene\_cluster | 30 | 48.5564304462 | 92 | 8.2e-19 |
| BAC76486.1 | putative\_NDP-hexose\_3-O-methyltransferase | BGC0000085 | Lankamycin\_biosynthetic\_gene\_cluster | 32 | 38.845144357 | 89 | 7e-18 |
| BAJ16473.1 | methyltransferase | BGC0000058 | FD-891\_biosynthetic\_gene\_cluster | 29 | 43.0446194226 | 89 | 9.1e-18 |
| AAO65792.1 | monensin\_3-O-methyl\_transferase | BGC0000100 | Monensin\_biosynthetic\_gene\_cluster | 36 | 25.4593175853 | 81 | 3.2e-15 |
| EFG04600.1 | Staurosporine\_biosynthesis\_methyltransferase\_StaMA | BGC0000826 | Staurosporine\_biosynthetic\_gene\_cluster | 28 | 48.031496063 | 79 | 7.2e-15 |
| ADC45521.1 | cyclopropane-fatty-acyl-phospholipid\_synthase | BGC0000093 | Meilingmycin\_biosynthetic\_gene\_cluster | 24 | 56.9553805774 | 75 | 1.8e-13 |
